# A new inclusive MLVA assay to investigate genetic variability of *Xylella fastidiosa* with a specific focus on the Apulian outbreak in Italy

**DOI:** 10.1038/s41598-020-68072-5

**Published:** 2020-07-02

**Authors:** Angelo Mazzaglia, Yaseen Jundi Rahi, Maria Claudia Taratufolo, Marta Tatì, Silvia Turco, Serena Ciarroni, Vincenzo Tagliavento, Franco Valentini, Anna Maria D’Onghia, Giorgio Mariano Balestra

**Affiliations:** 1 Dipartimento di Scienze Agrarie e Forestali (DAFNE), Università della Tuscia, 01100 Viterbo, Italy; 2 Phytoparasite Diagnostics s.r.l., 01100 Viterbo, Italy; 3 CIHEAM-Mediterranean Agronomic Institute of Bari, 70010 Valenzano, BA Italy

**Keywords:** Microbial ecology, Bacterial evolution, Bacteriology, Classification and taxonomy, Genetic markers, Haplotypes, Genetic variation

## Abstract

The Olive Quick Decline Syndrome by *Xylella fastidiosa* subspecies *pauca* is among the most severe phytopathological emergencies nowadays. In few years, the outbreak devastated olive groves in Apulia (Italy), potentially endangering the entire Mediterranean basin. This research aimed to develop a multiple locus VNTR analysis assay, a molecular tool to differentiate between populations of the pathogen. It has already been successfully applied to different *X. fastidiosa* subspecies from various plant hosts. The previously published TR loci, together with a set of new design, have been tested in silico on the genome of the Apulian De Donno strain. The resulting selection of 37 TR loci was amplified on the genomic DNAs of the Apulian strains AND from representatives of *X. fastidiosa* subspecies, and directly on DNA extracted from infected plants. The assay clearly discerned among subspecies or even sequence types (ST), but also pointed out variants within the same ST so as to provide more detailed information on the dynamics and pathogen diffusion pathways. Its effective application even on total DNAs extracted from infected tissues of different host plants makes it particularly useful for large-scale screening of infection and for the strengthening of containment measures.

## Introduction

*Xylella fastidiosa* is a Gram-negative phytopathogenic bacterium belonging to the *Xanthomonadaceae* family, which is able to infect and cause diseases on 563 plant species^1^. It is transmitted by several xylem sap-feeding insect species, especially sharpshooters, froghoppers (Hemiptera: Cicadellidae) and spittlebugs (Hemiptera: Cercopidea)^2^. The bacterium grows in the xylem of the host, where it actively multiplies and forms a biofilm slowly occluding xylem vessels, thus causing water stress and nutritional deficiencies^3^. *X. fastidiosa* causes a broad range of symptoms according to the infected host. The first disease attributable to this pathogen was the Pierce’s disease (PD) on grapevine. Over the years, many other diseases have progressively been reported on crops, ornamentals and woody plants^4^, such as Citrus Variegated Chlorosis (CVC)^5^, and several leaf scorch diseases e.g. almond leaf scorch (ALS). At present, relying on DNA-DNA hybridization and MLST data^2^, four *X. fastidiosa* subspecies have been classified, each one specific for a particular range of host plants and a native zone^6–8^. These subspecies are: (1) *fastidiosa*, which causes, among others, PD and ALS in North and Central America, (2) *sandyi* causing OLS in California, (3) *multiplex*, associated with scorch diseases of a wide range of trees in North and South America, and (4) *pauca*, mostly found in South America on citrus and coffee^9,10^. Two additional subspecies have been proposed: (1) *morus*, which includes isolates infecting mulberry^11^, and (2) *tashke*, from *Chitalpa tashkentensis* in southwestern USA ^12^. The evolutionary history and the geographical distribution of *X. fastidiosa* subspecies in the Americas indicate that the different subspecies evolved in geographic isolation, *X. fastidiosa* subsp. *multiplex* and subsp. *sandyi* in North America, subsp. *fastidiosa* in Southern-Central America and subsp. *pauca* in South America^2^. However, anthropogenic activities have introduced them into new areas. Indeed, the pathogen was reported in 2002 in Taiwan^13^, in 2013 in Italy^14^, in 2014 in Iran^15^, in 2015 in France^10^, in 2016 in Spain^16^, and in 2018 in Portugal^17^ and Israel^18^. All these new outbreaks raised up the need to accurately estimate the changes of genetic features to understand the dynamics and evolutionary process of populations, as well as the adaptation to different hosts and environments. In response to this, several molecular technologies have been tested on *X. fastidiosa*. These include non-sequence-based methods, such us restriction fragment length polymorphism (RFLP)^19^, randomly amplified polymorphic DNA (RAPD)^20^, and amplified fragment length polymorphism (AFLP)^21^, but with some limitations due to low reproducibility and potential homoplasy of alleles. Sequenced-based methods targeting specific regions such as 16S rDNA or 16S-23S internal transcribed spacer (ITS) were applied, with some success, as with the new subspecies *tashke* identified and proposed by Randall et al.^12^ using these approaches. It has been demonstrated that the introduction of foreign *X. fastidiosa* strains in new geographical areas and subsequent recombination with endemic strains may be relevant in increasing the genetic variability, shifting the target host and thus, inducing new crop diseases^22^. In this regard, multilocus sequence typing (MLST), based on the identification of nucleotide sequence differences in seven housekeeping genes, has been applied to study the evolution of *X. fastidiosa* and its subspecies^6, 23^. Through this method, it was proposed that the new subsp. *morus* originated by intersubspecific homologous recombination from *X. fastidiosa* subsp. *fastidiosa* and *X. fastidiosa* subsp. *multiplex*^11^. Likewise, MLST analysis of *X. fastidiosa* subsp. *pauca* isolated from coffee plants in Costa Rica and subsequently from olive trees in Italy was referred to the ST53 and provided important information about the origin of the outbreak^22,24,25^. However, despite being a powerful tool, MLST has some limits: relying on only seven core genome genes could be less performing in differentiating between very closely related strains and, moreover, it is not suitable for large scale and routine monitoring because of the costs of sequencing^4,10,26,27^. The whole genome sequencing is the most informative approach; however, although extremely powerful, this analysis requires highly skilled personnel, is time-consuming and costly, making it poorly suitable for fast and large-scale surveys^28^. Instead, tools based on markers with an adequate discrimination power like Simple-Sequence Repeat (SSR), also known as variable-number tandem repeat (VNTR), are a good compromise to analyse genetically homogeneous bacteria. The analysis of VNTR loci is the basis for multiple-Locus VNTR analysis (MLVA), where the number of repeats can be determined by PCR amplification using primers complementary to the well-conserved sequences flanking the tandem repeats. This method is rapid, easy to perform, inexpensive and highly reproducible^28–30^. Indeed, the MLVA analysis has often been adopted by microbiologists to study population structure of several human and animal bacterial pathogens, such as *Mycobacterium tuberculosis*, *Yersinia pestis*, *Staphylococcus aureus*, *Salmonella typhimurium*, and *Mycobacterium bovis*^31^. Among plant pathogens, several studies have already been carried out using SSR markers for *X. fastidiosa* genotyping. In 2001, Della Coletta Filho et al.^32^ described the efficacy of a set of 9 SSR markers in comparison with RAPD to evaluate genetic diversity of Brazilian strains of *X. fastidiosa* subsp. *pauca*, responsible for citrus variegated chlorosis (CVC) ; in 2005, Lin et al.^33^ used a genome-wide approach to design a new set of 34 SSR to estimate genetic diversity among 43 isolates of *X. fastidiosa* collected in California from grape, almond, citrus and oleander, obtaining significant results. A similar approach was used in 2007 by Montero-Astùa et al.^34^ on different plant species from Costa Rica, Brazil and USA in combination with other techniques to understand the relationship between strains. In 2014, Della Coletta-Filho et al.^35^ used MLVA to provide information on the genetic diversity of populations in sweet orange, as well as the consequences of vector transmission of *X. fastidiosa* on their structure. Another successful SSR genotyping has been proposed to analyse the seasonal and annual variation in genetic diversity of this bacterium in two almond orchards in California^36^. In the same year a combined use of SNP-based assay and multilocus SSR markers was attempted to assess the genetic diversity of *X. fastidiosa* subsp. *pauca* infecting citrus and coffee^37^. More recently, a new MLVA assay with 7 SSR markers was also used to demonstrate that *X. fastidiosa* subsp. *pauca* populations from coffee have higher genetic diversity and allelic richness compared with those from citrus^38^. These researches represented the starting point for this study, focused on *X. fastidiosa* subsp. *pauca*, which gained enormous attention after its detection on olive trees in Italy in 2013^39^ and its new pathogenic expression described as Olive Quick Decline Syndrome (OQDS)^40^. It represents an exemplary model of the introduction of an exotic pathogen in an area where a cultivated species, not coevolved with the pathogen, proves to be extremely susceptible to its attack. This, together with the transmission by non-host-specific xylem-feeding insects and the difficulty to control a pathogen living in the vascular system of the infected plant, has led to an impressive spread of the disease within few years. Despite massive efforts in containment measures, nowadays thousands of hectares of olive groves in Apulia are sevrely affected by the syndrome and the spread is still going on. Again, in this situation, a fine-tuned genotyping of the strains responsible for the outbreak is crucial to understand where the disease comes from, how it moves in the infected areas and to monitor if new variants would appear in this scenario or if the present type undergoes evolutionary forces that can lead to new variants. Today it is acknowledged that the strains infecting olive trees, but also other plant species in Italy, belong to the sequence type ST53, and the most plausible origin of the Olive Quick Decline Syndrome refers to strains infecting imported coffee plants (as ornamentals) from Costa Rica^25,39,41,42^. It is worth noting that the same ST53 was reported from imported plants in both France and the Netherlands^10,41^. Thus, we firstly aimed to check in silico the presence of the VNTR loci reported in the literature within the completely edited genome of the De Donno strain (accession $\hbox {n}^{\circ }$ CP020870). A further search for new VNTR loci was independently conducted on the same genome, aiming to obtain a final selection of markers to be used in a novel, inclusive MLVA assay capable to generate novel and deeper information about the genetic diversity of this subspecies, with specific reference to the Italian outbreak in Apulia.

## Results

### In silico analysis of VNTR loci from literature

The in silico check of the 50 TRs and related primers reported in literature on the genome of the De Donno strain of *X. fastidiosa* subsp. *pauca* showed several inconsistencies. First of all, numerous markers resulted to be the same, even if reported in the papers with different names and amplified with different primers. In some cases, the tandem repeat is also reported as reverse and opposite sequence. More specifically, SSR20 marker^32^ is the same as COSS1 marker^38^, SSR28 marker^32^ is the same as marker ASSR-14^33^, SSR30 marker^32^ is the same as marker OSSR-19^33^, SSR32 marker^32^ is the same as COSSR6 marker^38^, CSSR-17 marker^33^ is the same as COSSR3 marker^38^, OSSR-9 marker^33^ is the same as marker ASSR-20^33^, OSSR-14 marker^33^ is the same as CSSR45 marker^38^, OSSR-16 marker ^33^ is the same as CSSR-20 marker^33^, OSSR-17 marker^33^ is the same as CSSR-7 marker^33^, CSSR-18 marker^33^ is the same as GSSR-6 marker ^33^, and GSSR-12 marker^33^ is the same as CSSR42 marker^38^. These correspondences between markers from different studies and their respective positions along the De Donno genome are summarized in Table S1. In these cases only one pair of primers was chosen for the amplification, i.e. the ones whose sequences best fitted *X. fastidiosa* subsp. *pauca* strain De Donno (loci highlighted in yellow in Table S2). In addition to these duplications, several other discrepancies have been found in comparison to the De Donno genome, which are marked as nucleotides in red in Table S2. Specifically for markers from the study of Della Coletta-Filho et al.^32^, it was not possible to detect the reverse primer for SSR26 marker, SSR32 marker contains 2 different tandem repeats, both of 8 bp (the one reported is in green) and one SNP in the forward primer, for SSR36 and SSR40 markers, the TR was not retrievable even if their respective primers were detected with few differences, for both; finally, none of the two primers for the SSR34 marker amplification was found. Regarding the markers described by Lin et al.^33^, it was not possible to find the forward primer of OSSR-12 marker; OSSR-19 marker has an SNP in the reverse primer; only a single TR was found for CSSR-4, CSSR-6, GSSR-14, GSSR-15, GSSR-19, GSSR-20 markers; TR is absent for CSSR-12 and CSSR-13 markers; the primers of CSSR-16 marker show multiple annealing sequences; a different TR sequence and one SNP were found in the reverse primer for ASSR-16 marker, one SNP was found in the forward and one in the reverse primer of both ASSR-19 and GSSR-4 markers. No discrepancies were found in the 7 VNTR loci described in the paper of Francisco et al.^38^. After this check, several markers were discarded due to duplication, failure to detect the primer sequence or repeat sequence. Also, all the differences detected between the primer sequences and the corresponding pairing sequences on the De Donno genome were accordingly corrected (nucleotides in green in Table S2) for the synthesis of the primers for this study that are reported in Table 1.

**Table 1:** Primer pairs for the amplification of the selected 37 VNTR loci and sequence of the respective tandem repeats

TR	Locus	Forward primer	Reverse primer	TR sequence
1	SSR20	ATGAAGAAGCCAGGATACAT	GCTACACGTGCAACAAC	ATTGCTG
2	SSR21	AACACGGATCAAGCTCATG	GGAACACGCAATAGTAAGA	TGTTATC
3	SSR28	GTAACGCTGTTATCTCAAT	ATTACGCTTCTTATCGCTGT	GTGTGCCT
4	OSSR-9	TAGGAATCGTGTTCAAACTG	TTACTATCGGCAGCAGAC	TTTCCGT
5	OSSR-16	GCAAATAGCATGTACGAC	GTGTTGTGTATGTGTTGG	CTGCTA
6	OSSR-19	GCTGTGAACTTCCATCAATCC	GCAAGTAGGGGTAAATATGAC	CAGGATCA
7	OSSR-20	ATCTGTGCGGCGGTTCTG	CACTTGCGGCGTAGATACTTC	AGGATGCTA
8	CSSR-7	CACAGCGAACAGGCATTG	AGCAACCAAGACGGGAAC	CTGTGC
9	CSSR-10	GCAACCACAAAGCCGCAG	AGCACCTCTTAGCATCACTGG	CAATGA
10	CSSR-18	GTGCTTCCAGAAGTTGTG	GACTGTTCTCTTCGTTCAG	GCCAA
11	CSSR-19	TGCTGTGATTGGAGTTTTGC	TCAAACGAATCTGTCCATCAAG	TGGTGAG
12	ASSR-9	GGTTGTCGGGCTCATTCC	TTGTCACAGCATCACTATTCTC	CAAGTAC
13	ASSR-11	AGAGGCAACGCAGGAACAG	GTGAGTTATATCGGTGCAGCAG	ACGCATC
14	ASSR-12	TGCTCATTGTGGCGAAGG	CGCAACGTGCATTCATCG	GATTCAG
15	ASSR-16	TTAATCAACAACGCTTATCC	TCGCAGTAGCCAGTATGC	GCTCCA
16	ASSR-19	CGCCGACTGTCTATATGAC	TTCGTAGCAATGGCAATGTTG	ACAACG
17	GSSR_4	GCGTTACTGGCGACAAGC	GCTCGT(C)TCCTGACCTGTG	ATCC
18	GSSR_7	ATCATGTCGTGTCGTTTC	CAATAAAGCACCGAATTAGC	GGCAAC
19	COSSR6	TGCTGCGCGATAACCAAGT	CATCCAATCAGCCCTAACCT	GTGATGCG
20	CSSR45	ACAGACATCACCGGCATTG	AATGTCGCTGCCAATCCAT	CACACCGAGATGGAC
21	COSSR4	CAAGGTGACCGCTAGCCTAT	GCTGTCATTGGGTGATGC	CAATACAC
22	COSSR5	ACACTGACACAACAGCCACCA	AATGGTGGGTGTGATGGTTTC	CATACAGA
23	CSSR42	ATTACGCTGATTGGCTGCAT	GTTTCATTACGCGGAACAC	TGTTATC
24	TR4	CATACGGCAGTTCTGTGTCG	CGGGCAAGCTTTTCCCACCC	CAGCGCAT
25	TR5	ATTCCAAGATTTGCGAGTGG	ACGATTCGAACATGGAGGTA	TTCTAG
26	TR6	ACATCGGAGGTAGGCTGTGA	ATTGAAGACCCTTTTCAGCC	CGCTTAT
27	TR7	GGGTTGGGTCTTTTATTTGC	CATTGACTCTCAACCCTGCTAC	GCTGT
28	TR8	GCGGTTTGGTTGTATTGCTT	CTCACATCACGCACCGACGA	GACAGG
29	TR9	GGTGTGCCGTGTACATTGAG	TTGCCATCACCGACACCTCT	ATGATCTGA
30	TR10	CGTGCTGAAGTCTTGCTTGA	ACTTCACCCTACCCTGCATA	GTAACG
31	TR12	AGGGATATAGTGCCGCGATT	TTTTGTGGTCGAACGTGCGG	GGTGTGA
32	TR15	ATGCAGCGGTAGTCCCTCTA	CACGATGCCCACGTAGCAGC	GTGTCG
33	TR18	TGTCATGACCGTGCTTATGG	TGGTGGTCAAGGCAGCGG	CCGCCGCCGTAACCACCG
34	TR19	CTGCCTTGACCACCACCAC	ACAAAGCTCTCTGATCAATCAC	CCACTCCAGCTG
35	TR21	CAGGGTGTATGGCCTGAAGT	CCTACCATCCATGCAGCAAC	CAGCACAT
36	TR23	CAGGAGCCTCCATGAACAAT	AATGATCCTTGCTGGGTCAG	CTTCAAGAG
37	TR24	ATGGCCCAAACATACTCCAA	TGTTCATATCTTGGTCTCAT	GTCCTG

TRs 1–3 were obtained from Della Coletta-Filho et al., 2001; TRs 4–18 were obtained from Lin et al., 2005; TRs 19–23 were obtained from Francisco et al., 2015; TRs 24–37 are from this study.

### New VNTR loci identification

The searching procedure by Tandem Repeat Finder (TRF; https://tandem.bu.edu/trf/trf.html)^43^ has led to the identification of 25 VNTR loci, which have also undergone an in silico check to ascertain their position on the De Donno genome and to verify any correspondence with loci selected from literature. Again, this correspondence was found for 5 of them, which were consequently discarded. At the end, 45 total VNTR loci were selected (25 from the literature and 20 newly designed) to be used in the following experimental procedures.

### PCR amplification of VNTR loci

A first round of PCR was done using only genomic DNAs from two strains, the De Donno strain from olive and the V104 strain isolated from oleander, to validate the efficacy of the entire MLVA assay. The primer pairs related to newly designed VNTR loci (TR3, TR11, TR14, TR16, TR17 and TR25) produced multiple amplicons, indicating the presence of multiple pairing sites for at least one of the two primers and were consequently discarded. Also, SSR40 locus^32^ invariably produced a 133 bp amplicon, corresponding exactly to the sum of the only flanking regions, thus indicating the absence of the tandem repeat; whereas OSSR-2 locus^33^ invariably produced a 181 bp amplicon, smaller than the sum of the flanking regions; both were discarded. Table 1 shows the list of 37 loci and the related final primers used in the amplification step. According to the amplicon sizes obtained by capillary electrophoresis, the number of repeats per locus and per strain was calculated. Amplification failure was eventually coded as “0”. Thus, the haplotype of each individual was defined as the ordered sequence of 37 numbers, as reported in Table S3. It has to be noticed that the amplification of two DNAs obtained from infected tissues of *Prunus dulcis* and *Polygala myrtifolia* in the province of Lecce (Pd_Le2 and Pm_Le10) provided multiple bands in some loci. Due to these discrepancies, which cannot be properly coded as input data, both samples were excluded from the following analysis.

### Data analysis

**Figure 1:**
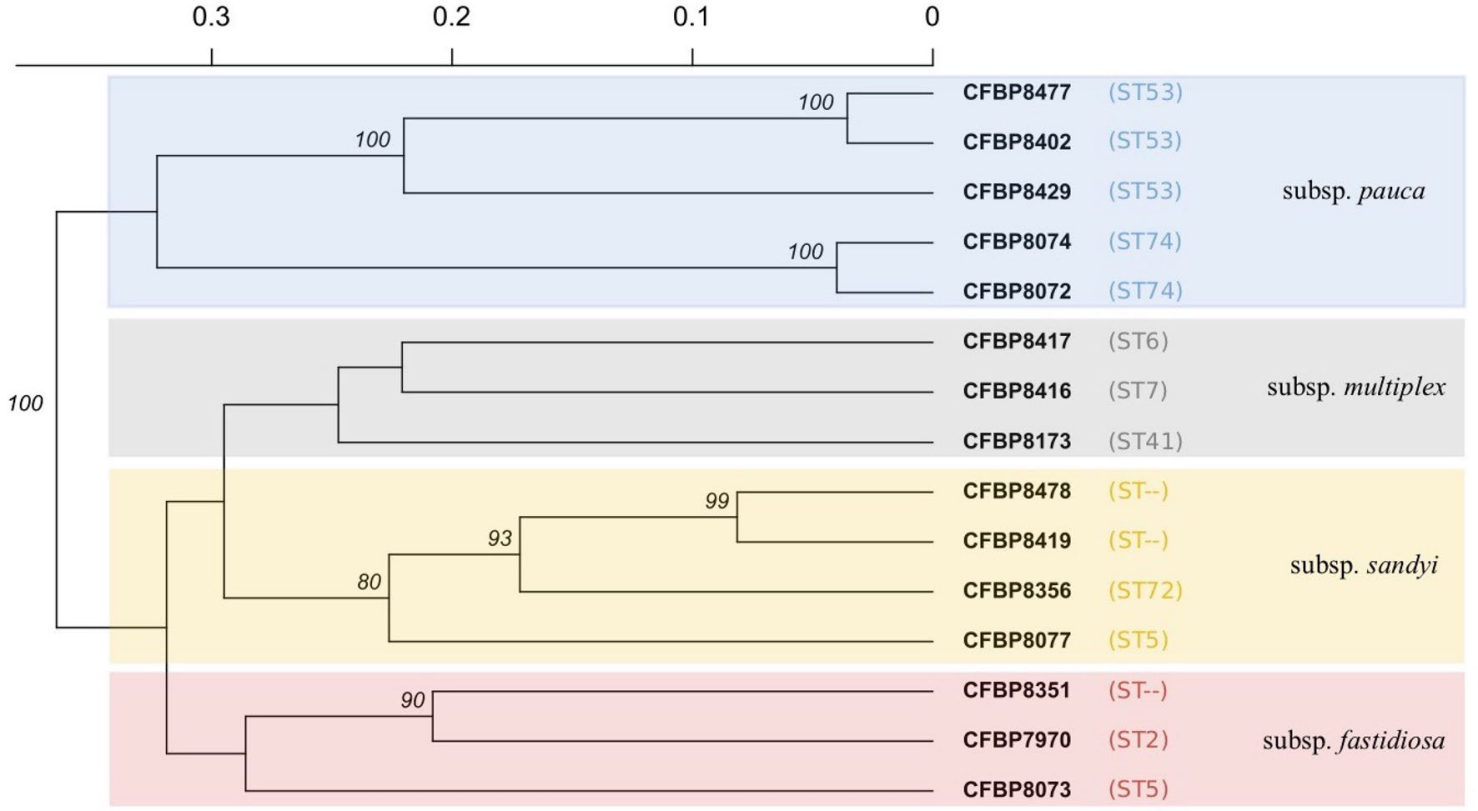
Hierarchical clustering of 15 *Xylella fastidiosa* strains from CFBP based on MLVA results and obtained using Bruvo’s genetic distance, UPGMA as agglomerative algorithm, and 1,000 bootstrap replications. Backgroud colors refers to subspecies. Only bootstrap percentages above 80% are reported.

**Figure 2:**
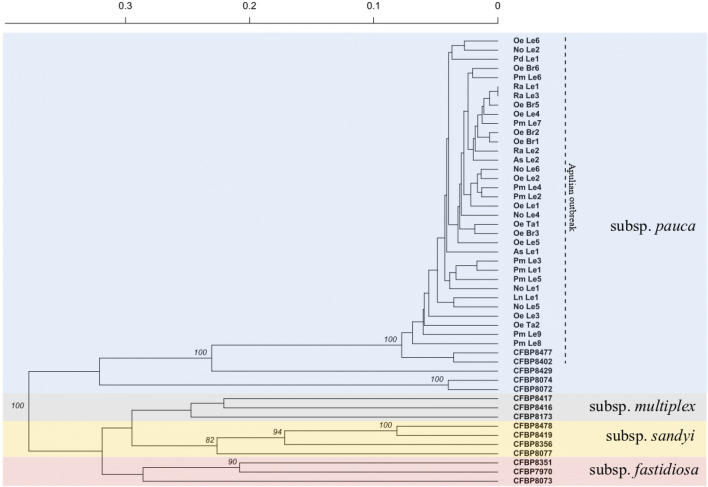
Hierarchical clustering of *Xylella fastidiosa* strains from both CFBP and from the Apulian oubreak, and of total DNAs from infected plants in Apulia, based on MLVA results and obtained using Bruvo’s genetic distance, UPGMA as agglomerative algorithm, and 1,000 bootstrap replications. Backgroud colors refers to subspecies. Only bootstrap percentages above 80% are reported.

**Figure 3:**
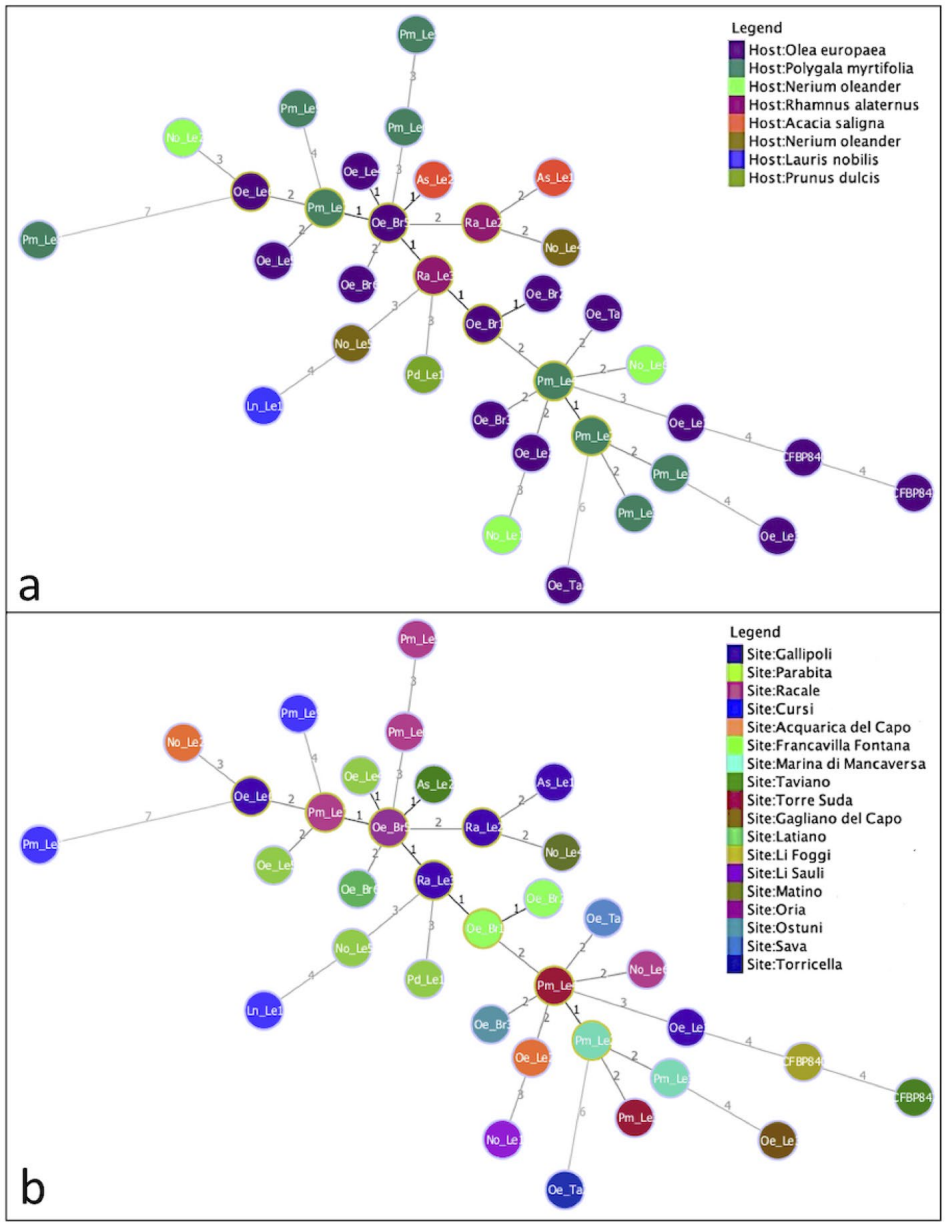
Minimum spanning tree (MST) of *Xylella fastidiosa* strains. (**a**) the color of the haplotypes corresponds to the host plant species; (**b**) the color of the haplotypes corresponds to their geographic origin at locality scale. Numbers on lines connecting two haplotypes represents the number of variable loci out of 37.

The haplotypes obtained by the amplification of 37 VNTR loci on a total of 51 DNAs are reported in Table S3, which includes the genomic DNAs extracted from 15 strains of the CFBP collection, 9 strains isolated in Apulia and 27 total DNAs as extracted from infected plant samples; the last 2 lines of the Table S3 reveal that multiple repeats have been detected in some loci of the samples Pd_Le2 and Pm_Le10, which were not considered in the subsequent analyses. The first run of data processing was carried out limited to the 15 strains from CFBP collection by hierarchical clustering using Bruvo’s distance (Fig. 1) to check the efficacy of the method to assess genetic differences between subspecies and sequence types (STs). Four main clusters were formed, each corresponding to one subspecies of *X. fastidiosa*. However, the strains of the same subspecies yet belonging to different STs are separated by genetic distances greater than those measured between strains belonging to the same sequence type, i.e. the two strains of subspecies *pauca* belonging to ST74 and two, out of three, belonging to ST53. Following the focus of the paper, the data obtained from VNTR loci amplification of DNAs extracted from strains isolated in Apulia and from DNAs extracted from infected tissues of different host plants in the same region were added for the second run of analysis. We were expecting that the very high number of loci analyzed here would highlight even minimal differences between samples. Indeed, among all the individuals, only 2 haplotypes obtained from samples of infected *Rhamnus alaternus* plants in the same geographic location were found completely identical to each other along the 37 loci. The hierarchical clustering of all the samples (Fig. 2) maintains the same structure as the previous dendrogram, with the addition of all the Apulian specimens to the subspecies *pauca* cluster. Within this group, the CFBP8429 strain, isolated from *Coffea arabica* plants in Angers (France) in 2015 and reported in the CFBP database as belonging to the ST53, results significantly separated from all the others ST53 from Italy. Lastly, the 38 DNAs of Apulian origin were analysed independently to better appreciate their relationships, given that their reciprocal genetic variability turned out to be extremely low. Indeed, an identical number of repetitions was obtained in as many as 18 loci out of 37 (48%), and, among the remaining 19 loci, 11 changed sporadically in few samples, whilst only 7 loci (TR7, TR8, TR12, OSSR-16, OSSR19, ASSR-16, and COSSR-4) showed frequent variations in the numbers of repeats. Table S4 reports the position of the latter loci, amplified by the primers used in this study, along with the genome of De Donno strain of *X. fastidiosa* subsp. *pauca*, and the incidental inclusion of coding sequences. In Fig. 3, the Minimum Spanning Trees (MST) illustrate how the haplotypes of these samples are linked to each other at best. The MSTs reveal that no specific correlation of the haplotypes with the species of host plants from which they were obtained (Fig. 3a) can be inferred, nor with the specific geographic origin (Fig. 3b).

## Discussion

*Xylella fastidiosa* is one of the most feared bacterial plant diseases nowadays. Several biological features make its management very challenging, first and foremost for its ability to colonize an astonishing number of plant hosts, often symptomless, as well as for the difficulty in its isolation and detection. A crucial issue to better understand the spread dynamics is the genotyping of the pathogen, which can be carried out by different molecular techniques. MLST is the method of choice for *Xylella fastidiosa* genotyping at subspecies level and beyond ^44^, based on the detection of SNPs included in seven constitutive genes^45^. By this method, it is possible to further categorize the six subspecies of *Xylella fastidiosa* in 87 different sequence types (STs)^8,22,23,26,46^. The protocol entails the specific PCR amplification of the genes using DNA extracted from both cultured strains and infected plant tissues, followed by Sanger sequencing, preferably in both strand directions^44^, that take time to get the final results. This might be a limit if large numbers of samples are to be analysed, as in epidemic monitoring^4,10,26,27^. According to this method and to our knowledge, all the strains related to the Apulian outbreak in Italy analysed so far are univocally assigned to the genotype ST53 of *X. fastidiosa* subsp. *pauca* ^11,25,47^. MLVA has proven suitable in genotyping *Xylella* as well, with a specific focus on assessing genetic variability among strains involved in local outbreaks. This distinctive feature is testified in numerous studies on different host plants such as grapevine^48^, orange^35^, almond tree^36^ and coffee^38^. Only recently, a first study using a panel of 12 SSRs on *X. fastidiosa* subsp. *pauca* strains from olive trees in Brazil was published^46^. This study explored the potential of this method in detecting subtle genetic differences among a selection of samples from the *Xylella* outbreak in Apulia, relying on its high sensitivity coupled with a mere amplicon dimension measurement. To our knowledge, a new inclusive MLVA assay was developed for the first time, and applied to this scenario. It’s worth emphasizing the importance of a preliminary in silico analysis of markers when obtained from multiple literature sources. Indeed, the screening carried out here revealed that several loci from previous independent studies were the same. This leads to the first noteworthy consideration that, even if lots of repeats can be found along a whole bacterial genome, the effective loci with features suitable for proper genotyping are much less common and numerous. Moreover, a clearer classification of markers to avoid misunderstanding and confusion in their use appears is highly needed. As an example, in Safady et al.^46^ twelve markers from literature have been used^33,38^; however, our in silico analysis demonstrated that two of them, i.e. CSSR42 and GSSR12 loci, refer to the same repetition, giving the same result. This kind of misunderstanding can negatively affect results and possibly lead to incorrect conclusions. In silico screening and the first round of PCR testing have almost halved a large number of potential markers (50 from literature and 25 newly designed) to a selection of 37 affordable TR loci. However, this is still a conspicuous set - thoroughly tested - to detect genetic differences among *X. fastidiosa* DNAs from the Apulian outbreak. Two analytical approaches were independently applied to haplotype data according to their peculiarities. The Bruvo’s genetic distance^49^ is particularly suitable for genetic markers as tandem repeats because it accounts for repeat length into calculation and is not sensitive to ploidy levels^50–52^. For these reasons it was used to build the dendrograms of Figs. 1 and 2. The approach proposed by Francisco et al.^53^, in which the efficacy of eBURST algorithm^54^ was reinforced with additional rules to better elucidate possible patterns of evolution, was used to analyse the Apulian dataset in detail. Although MLVA is not primarily aimed to ascertain phylogeny, due to its inherent sensitivity in appreciating minimal differences between individuals, this assay has proven proficient to correctly cluster subspecies of *Xylella fastidiosa* under the recognized phylogeny. However, the use of few representatives doesn’t allow to draw consistent conclusions. When compared with MLST categorization, this assay seems to have a higher discriminatory power, as highlighted by the distinction within STs in the results. Therefore, it is of particular importance that CFBP 8429 strain, isolated from a coffee plant intercepted in Angers (France) in 2015, and belonging to the ST53 (as reported in CFBP database), showed substantial differences, in MLVA analysis, in the number of repetitions compared to the other samples of the same ST53. This led to its independent positioning in hierarchical clustering, with 100% bootstrap support, and in MST. However, we cannot exclude that this discrepancy could be related to misidentification of its ST, so that a larger comparison with isolates of the same subspecies from other geographic regions is recommendable to validate this hypothesis. In this study, the MLVA assay was steadily successful in ascertaining differences within single ST, differentiating between almost all the samples. This could make its utilization valuable in scenarios like the Apulian outbreak, where a highly clonal population of *X. fastidiosa* subsp. *pauca*, belonging only to ST53 and whose origin is probably attributable to a single infection, is under investigation. In our data, such clonality is widely confirmed and information capable of differentiating between individuals are relegated to a few loci. As to these most variable loci, looking at their correspondence in the genome sequence of the De Donno strain, it is noticeable that in five out of seven the tandem repeats are included within classified or hypothetical proteins, highlighting their role for the adaptation ability of the pathogen. From a practical point of view, those VNTR loci could be selected to arrange a further MLVA assay capable to resolve close genetic relationships in this scenario. Once their robustness is confirmed, they could be multiplexed to obtain a final assay for large-scale monitoring. However, the small differences revealed within ST53 strains in this analysis don’t show evidence for specific relationships with the species of the host plant or with the geographic origin of the strains. This has not to be necessarily meant as a failure of the method. Most probably, these differences account for the first signal of random variability within the *Xylella* population in Apulia; they do not reflect yet the effect of any evolutionary pressure toward host-specific variants, nor to the effect of spatial separation toward local independent populations. This is consistent with the multi-host nature of the pathogen and the relatively short time lapse since the first outbreak. Since *Xylella fastidiosa* is very “fastidious” and time-consuming for isolation and culturing, MLVA assay developed in this study has, similarly to other molecular techniques, the significant advantage to eventually screen the DNA extracted straight from the infected plant material without losing reliability. In this respect, we also postulate that this method could diagnose infections by multiple genotypes in the same plant tissue, such as for the two samples Pd_Le2 and Pm_Le10, in which some loci showed simultaneously multiple amplicons, corresponding to different numbers of repetitions. In conclusion, all the results seem to indicate that this novel MLVA assay has the potential to become a valuable method for thorough monitoring of the Italian outbreak of *Xylella fastidiosa* subsp. *pauca*, as well as for any other *Xylella fastidiosa* epidemics.

## Methods

All the 24 strains analysed in this study are reported in Table 2, where subspecies, host plant, geographic origin, time of isolation, and ST classification are also indicated. Fifteen strains (marked with ^§^) were sourced from the CIRM-CFBP (Collection Française de Bactéries associées aux Plantes). These strains were grown on Buffered Charcoal-Yeast Extract (BCYE) medium at 25 °C for 3–4 weeks; 100 mg of bacterial cells were then collected, and DNA was extracted with the Nucleospin Plant kit (Macherey Nagel) according to the manufacturer’s instructions. Similarly, 9 strains (marked with *), were isolated from different host plants in Apulia and their genomic DNAs were extracted from freshly grown strains at CIHEAM-Mediterranean Agronomic Institute of Bari (CIHEAM-IAMB). The remaining 27 samples (marked with $^{\circ }$) are instead constituted by total DNAs extracted straight from tissues of plants whose infection by *X. fastidiosa* was previously assessed. These include DNAs from different host plants in various locations in Apulia, i.e. in the provinces of Lecce, Taranto, and Brindisi (Fig. S1^55,56^). All DNAs were checked by q-PCR to confirm their belonging to *X. fastidiosa* according to the protocol described in Harper et al.^57^.

**Table 2:** List of *X. fastidiosa* strains analysed in this study and details about thier subspecies, host plant, geographic origin, time of isolation, and ST classification

Sample	Subspecies	Host	Country (Region)	Province	Year	ST
CFBP8073^§^	*fastidiosa/sandyi*	*Coffea canephora*	Mexico	Mexico	2012	ST75
CFBP7970^§^	*fastidiosa*	Grapevine	USA (Florida)	Florida	1987	ST2
CFBP8351^§^	*fastidiosa*	*Vitis vinifera* L.	USA (California)	Fresno	1993	–
CFBP8077^§^	*sandyi*	*Nerium oleander*	USA (California)	Orange	1995	ST5
CFBP8356^§^	*sandyi*	*Coffea arabica*	France	(intercepted)	2015	ST72
CFBP8419^§^	*sandyi*	*Coffea arabica*	Costarica	Costarica	2015	–
CFBP8478^§^	*sandyi*	*Coffea arabica*	France	(intercepted)	2015	–
CFBP8173^§^	*multiplex*	*Prunus* sp.	USA (Georgia)	Georgia	1983	ST41
CFBP8416^§^	*multiplex*	*Polygala myrtifolia*	France (Corsica)	Propriano	2015	ST7
CFBP8417^§^	*multiplex*	*Spartium junceum*	France (Corsica)	Alata	2015	ST6
CFBP8429^§^	*pauca*	*Coffea arabica*	France (Loira)	Angers	2015	ST53
CFBP8072^§^	*pauca*	*Coffea arabica*	Equador	Equador	2012	ST74
CFBP8074^§^	*pauca*	*Coffea arabica*	Equador	Equador	2012	ST74
CFBP8402^§^	*pauca*	*Olea europaea*	Italy (Apulia)	Lecce	2014	ST53
CFBP8477^§^	*pauca*	*Olea europaea*	Italy (Apulia)	Lecce	2015	ST53
Oe_Le1*	*pauca*	*Olea europaea*	Italy (Apulia)	Lecce	2014	ST53
No_Le1*	*pauca*	*Nerium oleander*	Italy (Apulia)	Lecce	2016	ST53
Oe_Le2*	*pauca*	*Olea europaea*	Italy (Apulia)	Lecce	2017	ST53
No_Le2*	*pauca*	*Nerium oleander*	Italy (Apulia)	Lecce	2017	ST53
Oe_Le3*	*pauca*	*Olea europaea*	Italy (Apulia)	Lecce	2017	ST53
Pm_Le1*	*pauca*	*Polygala myrtifolia*	Italy (Apulia)	Lecce	2017	ST53
Pm_Le2*	*pauca*	*Polygala myrtifolia*	Italy (Apulia)	Lecce	2017	ST53
Pm_Le3*	*pauca*	*Polygala myrtifolia*	Italy(Apulia)	Lecce	2017	ST53
Pm_Le4*	*pauca*	*Polygala myrtifolia*	Italy(Apulia)	Lecce	2017	ST53
Oe_Le4$^{\circ }$	*pauca*	*Olea europaea*	Italy (Apulia)	Lecce	2018	ST53
Pm_Le5$^{\circ }$	*pauca*	*Polygala myrtifolia*	Italy (Apulia)	Lecce	2018	ST53
Pd_Le1$^{\circ }$	*pauca*	*Prunus dulcis*	Italy (Apulia)	Lecce	2018	ST53
Ra_Le3$^{\circ }$	*pauca*	*Rhamnus alaternus*	Italy(Apulia)	Lecce	2018	ST53
Ra_Le1$^{\circ }$	*pauca*	*Rhamnus alaternus*	Italy (Apulia)	Lecce	2018	ST53
Pm_Le6$^{\circ }$	*pauca*	*Polygala myrtifolia*	Italy (Apulia)	Lecce	2018	ST53
No_Le6$^{\circ }$	*pauca*	*Nerium oleander*	Italy(Apulia)	Lecce	2018	ST53
Oe_Le5$^{\circ }$	*pauca*	*Olea europaea*	Italy (Apulia)	Lecce	2018	ST53
Oe_Br5$^{\circ }$	*pauca*	*Olea europaea*	Italy(Apulia)	Brindisi	2018	ST53
Oe_Br1$^{\circ }$	*pauca*	*Olea europaea*	Italy (Apulia)	Brindisi	2018	ST53
Oe_Br2$^{\circ }$	*pauca*	*Olea europaea*	Italy (Apulia)	Brindisi	2018	ST53
Oe_Br6$^{\circ }$	*pauca*	*Olea europaea*	Italy (Apulia)	Brindisi	2018	ST53
Oe_Br3$^{\circ }$	*pauca*	*Olea europaea*	Italy (Apulia)	Brindisi	2018	ST53
Oe_Ta1$^{\circ }$	*pauca*	*Olea europaea*	Italy (Apulia)	Taranto	2018	ST53
Oe_Ta2$^{\circ }$	*pauca*	*Olea europaea*	Italy (Apulia)	Taranto	2018	ST53
Oe_Le6$^{\circ }$	*pauca*	*Olea europaea*	Italy (Apulia)	Lecce	2018	ST53
As_Le1$^{\circ }$	*pauca*	*Acacia saligna*	Italy (Apulia)	Lecce	2018	ST53
Ra_Le2$^{\circ }$	*pauca*	*Rhamnus alaternus*	Italy (Apulia)	Lecce	2018	ST53
As_Le2$^{\circ }$	*pauca*	*Acacia saligna*	Italy (Apulia)	Lecce	2018	ST53
Pm_Le7$^{\circ }$	*pauca*	*Polygala myrtifolia*	Italy (Apulia)	Lecce	2018	ST53
No_Le4$^{\circ }$	*pauca*	*Nerium oleander*	Italy (Apulia)	Lecce	2018	ST53
No_Le5$^{\circ }$	*pauca*	*Nerium oleander*	Italy (Apulia)	Lecce	2018	ST53
Ln_Le1$^{\circ }$	*pauca*	*Laurus nobilis*	Italy (Apulia)	Lecce	2018	ST53
Pm_Le8$^{\circ }$	*pauca*	*Polygala myrtifolia*	Italy (Apulia)	Lecce	2018	ST53
Pm_Le9$^{\circ }$	*pauca*	*Polygala myrtifolia*	Italy (Apulia)	Lecce	2018	ST53
Pd_Le2$^{\circ }$	*pauca*	*Prunus dulcis*	Italy (Apulia)	Lecce	2018	ST53
Pm_Le10$^{\circ }$	*pauca*	*Polygala myrtifolia*	Italy (Apulia)	Lecce	2018	ST53

Strains marked with ^§^ were obtained from CFBP collection, strains marked with * were isolated from plants in Apulia, whilst $^{\circ }$ indicates total DNAs extracted from infected plant tissues.

### In silico analysis of Tandem Repeats previously reported on *Xylella fastidiosa*

Molecular typing of *X. fastidiosa* by VNTR markers was already been carried out in the past with significant results. In this study, 9 upstream markers from Della Coletta-Filho et al.^32^, 34 from Lin et al.^33^ and 7 from Francisco et al.^38^ have been considered. The presence of these 50 markers and their respective primers was in silico checked on the completely edited genome of the strain “De Donno” of *X. fastidiosa* subsp. *pauca*, deposited in GenBank (accession number: CP020870), using Nucleotide BLAST (for the tandem repeats) or Primer BLAST (for the respective primers) tools in NCBI website.

### New VNTR loci identification and primer design

In the meanwhile, a new search, aimed to identify potential new markers to be added to the analysis, was carried out on the same genome using the TRF program^43^ set with the following parameters: 2 matches, 7 mismatches, 7 indels as alignment Parameters; 50 as Minimum Alignment Score; 250 as Maximum Period Size. A further selection of the results obtained was made imposing the following parameters :> 5 as Period Size, > 2 as Copy Number; > 90% as Percent Matches, Consensus Size as Period Size. This procedure led to the identification of 25 new Tandem Repeats loci for which suitable primers in respective flanking regions were designed using Primer3 with default parameters.

### PCR amplification of VNTR loci

All VNTR loci were amplified with single PCR reactions using the primer pairs reported in Table 1. Each reaction contained 12.5 $\upmu$l of GoTaq G2 Green Master Mix Master Mix 2x (Promega Corporation, USA), 1 $\upmu$l of DNA sample (40 ng), 1 $\upmu$l of forward primer and 1 $\upmu$l of reverse primer (10 $\upmu$M final concentration), 9.5 $\upmu$l of molecular grade SDW to the final volume of 25 $\upmu$l. The PCR amplifications were performed with a C1000 thermocycler (Biorad Laboratories Inc., Ca., USA). The following parameters were used for the TR loci identified in this study: initial denaturation for 5 min at 95 $^{\circ }$C, 40 cycles of denaturation for 30 s at 95 $^{\circ }$C, annealing for 30 s at temperatures ranging from 47.9 $^{\circ }$C to 58 $^{\circ }$C according to primers requirements, and extension for 36 s at 72 $^{\circ }$C, plus a final elongation step for 5 min at 72 $^{\circ }$C. For the primers obtained from literature review ^32,33,38^ the respective protocols were followed.

### Capillary electrophoresis

The QIAxcel capillary electrophoresis system (QIAGEN, Milan, Italy) was used to estimate precisely the size of the amplicons. The DNA High Resolution cartridge was used for all samples and the OM800 method was run, as recommended, to achieve maximum accuracy (2-3 bp maximum error) with amplicons ranging in size from 200 to 500 bp. No template controls (SDW) and size markers were included in each run. The results were analysed and interpreted using the ScreenGel v.1.6.0 software (QIAGEN), which gives accurate estimation of both size and concentration of amplicons. Then, the number of tandem repeats at each VNTR locus was calculated by subtracting the flanking regions size from the amplicon size and dividing the remaining by the repeat unit length. In case of loci with a truncated final repeat, the copy number was rounded down to the previous integer. To validate the robustness of the results, the entire PCR and capillary electrophoresis procedure was performed at least twice per sample. To check the accuracy of tandem repeats calculations, a random selection of amplicons was Sanger sequenced, as well as for each case of disputable TR number attribution. A final data matrix (Table S3) of 51 samples and 37 numbers of TR for each locus was produced.

### Data processing

The string of integers obtained as above constitutes the haplotype of each strain under investigation. These were reciprocally compared using independently two analytical approaches suitable for this type of data, the hierarchical clustering and the goeBURST algorithm. Data were analysed in two steps: first, only the 15 strains from the CFBP collection were included to check the effectiveness of the method in maintaining the correct subspecies structure and in detecting differences among Sequence Types (STs); then, the remaining 34 DNAs, all related to the Apulian outbreak, were added to assess their relative positioning and grouping.

#### Hierarchical clustering

The data matrix was imported into R version 3.4.4 (R Core Team, 2018). The genetic distance among individuals was calculated using Bruvo’s distance^49^, and bootstrapped using the poppr *bruvo.boot()* function with a cut-off threshold of 80%. The hierarchical clustering was then obtained by *hclust()* function of the R package stats (R Core Team, 2018) using UPGMA as agglomerative algorithm. The final dendrogram was visualized with the R package factoextra version 1.0.5^58^.

#### Globally optimized eBURST algorithm (goeBURST–Phyloviz)

The software Phyloviz 2.0^59^ was used for the goeBURST analysis and to build the MST, a tree in which the sum of the distances among all the isolates, as represented by data, is the shortest possible.

## Supplementary information

Supplementary information 1

Supplementary information 2

Supplementary information 3

Supplementary information 4

Supplementary information 5
