# Supplementary material for: A new inclusive MLVA assay to investigate genetic variability of Xylella fastidiosa with a specific focus on the Apulian outbreak in Italy
Source: Sci Rep. 2020 Jul 2;10:10856. doi: 10.1038/s41598-020-68072-5 (PMC7331650; doi:10.1038/s41598-020-68072-5)
Supplement: Supplementary file 1 — Supplementary information 1 [file 41598_2020_68072_MOESM1_ESM.pdf]

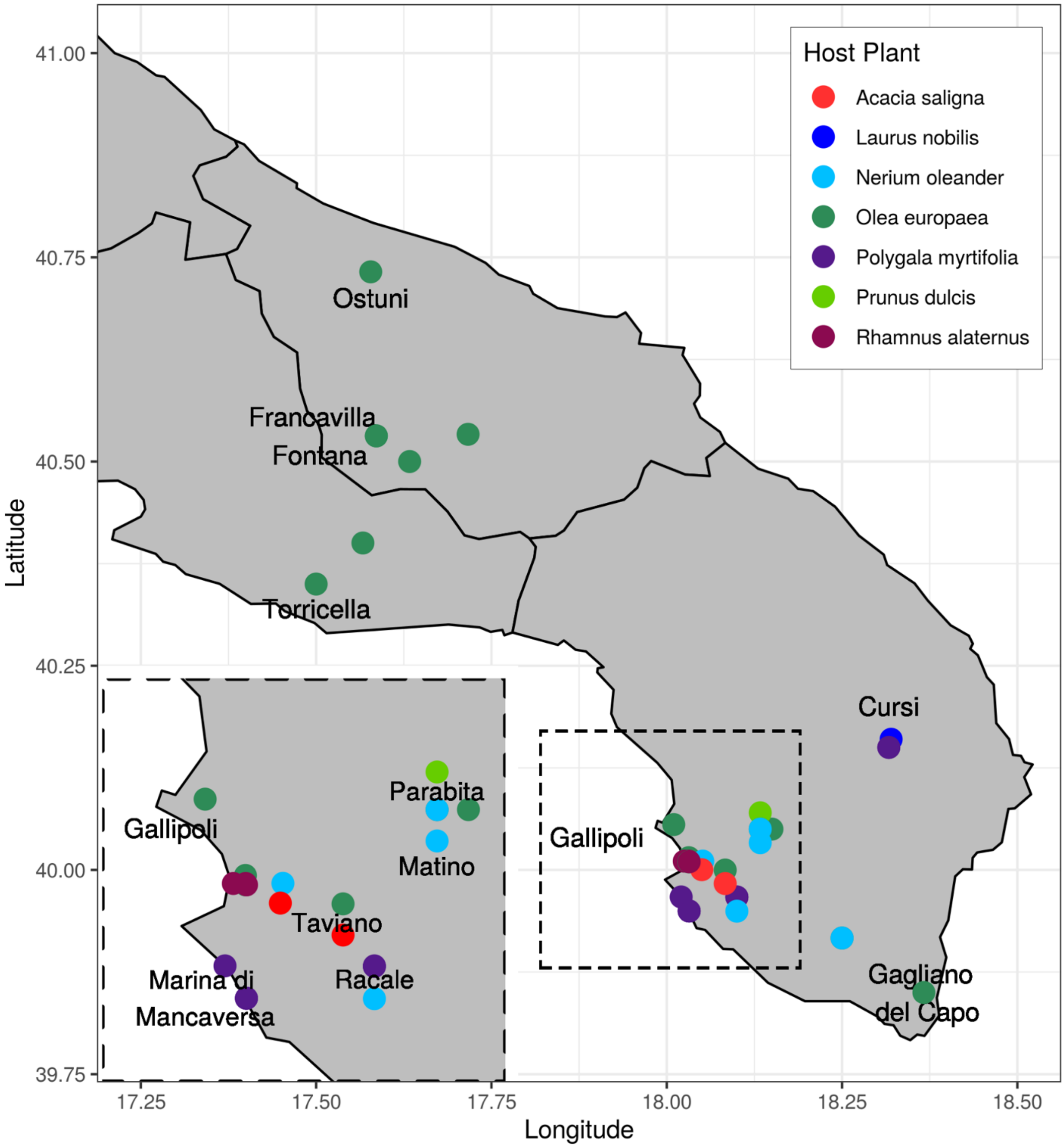

Figure S1. Map of sites in Apulia, Italy, where the samples were collected from different host plant species. The map was generated using the R packages *maps* (https://cran.r-project.org/package=maps) and *ggplot2* (https://ggplot2.tidyverse.org), version 3.3.0.
