# Supplementary material for: A new inclusive MLVA assay to investigate genetic variability of Xylella fastidiosa with a specific focus on the Apulian outbreak in Italy
Source: Sci Rep. 2020 Jul 2;10:10856. doi: 10.1038/s41598-020-68072-5 (PMC7331650; doi:10.1038/s41598-020-68072-5)
Supplement: Supplementary file 2 — Supplementary information 2 [file 41598_2020_68072_MOESM2_ESM.pdf]

VNTR locus	Reference	Forward primer	Reverse primer	Amplicon (bp)	TR sequence (as in references)	Correspondence to the De Donno genome sequence		
						TR sequence	Position	Direction
SSR20*	1	ATGAAGAAGCCAGGATACAT	GCTACACGTGCAACAAC	163	ATTGCTG	ATTGCTG	18360-18522	plus/plus
COSS-1	3	GAAACAAGATGGCGGTTGC	CATTTAAACGGGCGGCATA	292	ATTGCTG		18185-18476	plus/plus
SSR28*	1	GCAACGCTGTTATCTCAAT	ATTACGCTTCTTATCGCTGT	188	GTGTGCCT	AGGCACAC	1888166-1888353	plus/minus
ASSR-14	2	TTGACTCAAGGAATAAAAC	GAAAAGAGTGTCAATACG	408	CTGCGTGC		1888099-1888506	plus/minus
SSR30	1	TACGCTGCACCTGTCTG	CTGTGAACTTCCATCAATCC	231	TGATCCTG	CAGGATCA	1417984-1418214	plus/minus
OSSR-19*	2	GCTGTGAACTTCCATCAATCC	GCAAGTAGGGGTAAATGTGAC	290	CAGGATCA		1417983-1418272	plus/plus
SSR32	1	AGATGAACCTCGCCAC	GTACTCATCTGCGATGG	144	CTGATGTG	GTGATGCG	2480814-2480957	plus/plus
COSSR-6*	3	TGCTGCGCGATAACCAAGT	CATCCAATCAGCCCTAACCT	317	GTGATGCG		2480671-2480987	plus/plus
OSSR-9*	2	TAGGAATCGTGTTCAAACTG	TTACTATCGGCAGCAGAC	229	TTTCCGT	ACGGAAA	2301435-2301663	plus/minus
ASSR-20	2	TTACTATCGGCAGCAGACG	TGAAGCAATGGTGGATTTAGG	246	ACAGAAA		2301435-2301680	plus/plus
OSSR-14	2	GGCGTAACGGAGGAAACG	ATGAACACCCGTACCTGG	280	TGATCCATCCCTGTG	GTCCATCTCGGTGTG	129249-129528	plus/plus
CSSR-45*	3	ACAGACATCACCGGCATTG	AATGTCGCTGCCAATCCAT	319	CACACCGAGATGGAC		129332-129650	plus/minus
OSSR-16*	2	GCAAATAGCATGTACGAC	GTGTTGTGTATGTGTTGG	383	CTGCTA	CTGCTA	1779830-1780212	plus/plus
CSSR-20	2	GGTATCGCCTTTGGTTCTGG	GACAACCGACATCCTCATGG	379	GTAGCA		1779780-1780158	plus/minus
OSSR-17	2	AGTACAGCGAACAGGCATTG	AGCAACCAGGACGGGAAC	239	TGCCTG	CTGTGC	572633-572871	plus/plus
CSSR-7*	2	CACAGCGAACAGGCATTG	AGCAACCAAGACGGGAAC	237	CTGTGC		572635-572871	plus/plus
CSSR-17	2	AGAAGTATTCGCTACGCTACG	GGTGATGATTCAGTTGGTGTTG	151	CTGATGTG	CAGCACAT	899115-899265	plus/minus
COSSR-3	3	AAGTATTCGCTACGCTACGC	GTGTGTTATGTGTGCCATTCGT	300	CTGATGTG		898964-899263	plus/minus
TR_21*	this study	CAGGGTGTATGGCCTGAAGT	CCTACCATCCATGCAGCAAC	174	CAGCACAT		899148-899321	plus/plus
CSSR-18*	2	GTGCTTCCAGAAGTTGTG	GACTGTTCTCTTCGTTCAG	254	GCCAA	GCCAA	404278-404531	plus/plus
GSSR_6	2	TGTTCTCTTCGTTCAGCCAAGC	CGCAGCAGAGCAGCAGTG	209	CTTGT		404320-404528	plus/minus
GSSR_12	2	TTACGCTGATTGGCTGCATTG	GTCAAACACTGCCTATAGAGCG	335	TATCTGT	TGTTATC	52234-52568	plus/plus
CSSR-42*	3	ATTACGCTGATTGGCTGCAT	GTTTCATTACGCGGAACAC	285	TGTTATC		52233-52517	plus/plus

Table S1. Different VNTR loci from literature referring to the same locus according to the *in silico* comparison with the genome sequence of *Xylella fastidiosa* subsp. *pauca* strain De Donno (accession n° CP020870). The primers chosen for this study are indicated by * in the first column. References: 1) Della Coletta-Filho *et al.*, 2001; 2) Lin *et al.*, 2005.; 3) Francisco *et al.*, 2017.
