# Supplementary material for: A new inclusive MLVA assay to investigate genetic variability of Xylella fastidiosa with a specific focus on the Apulian outbreak in Italy
Source: Sci Rep. 2020 Jul 2;10:10856. doi: 10.1038/s41598-020-68072-5 (PMC7331650; doi:10.1038/s41598-020-68072-5)
Supplement: Supplementary file 3 — Supplementary information 3 [file 41598_2020_68072_MOESM3_ESM.pdf]

LOCUS	FORWARD PRIMER	REVERSE PRIMER	TR SEQUENCE
**Coletta-Filho *et al.*, 2001**			
SSR20	ATGAAGAAGCCAGGATACAT	GCTACACGTGCAACAAC	ATTGCTG
SSR21	AACACGGATCAAGCTCATG	GGAACACGCAATAGTAAGA	TGTTATC
SSR26	CTGTGATCGGTGAATTGA	TCAAGCACACTTCCTACG	GTGTGTGA
SSR28	GC(T)AACGCTGTTATCTCAAT	ATTACGCTTCTTATCGCTGT	GTGTGCCT
SSR30	TACGCTGCAC(G)CTGTCTG(T)	CTGTGAACTTCCATCAATCC	TGATCCTG
SSR36	ATGTCACTCAGGTCAGG	CAGAACCACCGACTG(CTCT)	TGTTGGGG
SSR40	ACCT(G)TGACGACGGATG	TAGGAACTGCTGCTACTGAT	GAAGGCGTA
SSR32	AGATGAACC(-)TCGCCAC	GTACTCATCTGCGATGG	CTGATGTG (GTGATGCG)
SSR34	TGATAGAACTGTTTGACGCATTTG	TCGGGAAGTTTGGGGTGAC	(TTGGGTAG)/(TTGGGTAA)
**Lin *et al.*, 2005**			
OSSR-2	TTGCTTCACCATTAGCCTTATC	GGCCGTACAGGACCGATC	ATG
OSSR-9	TAGGAATCGTGTTCAAACTG	TTACTATCGGCAGCAGAC	TTTCCGT
OSSR-12	ACAGTCTGTGTCCGCAATTTG	CAGGCGCAGATAGCATTGATC	AGAGGGTAT
OSSR-14	GGCGTAACGGAGGAAACG	ATGAACACCCGTACCTGG	TGA(G)TCCATCC(T)CT(G)GTG
OSSR-16	GCAAATAGCATGTACGAC	GTGTTGTGTATGTGTTGG	CTGCTA
OSSR-17	AGT(C)ACAGCGAACAGGCATTG	AGCAACCAG(A)GACGGGAAC	TGCCTG
OSSR-19	GCTGTGAACTTCCATCAATCC	GCAAGTAGGGGTAAATG(A)TGAC	CAGGATCA
OSSR-20	ATCTGTGCGGCGGTTCTG	CACTTGCGGCGTAGATACTTC	AGGATGCTA
CSSR-4	AACCCA(C)ATTCTTT-(G)TAATATGTG	TTGCAGCATTAGATATTTGAG	TGCC(A)
CSSR-6	CGCACTGTCATCCATTT(C)AATC	GCTGCTTCATCTAGACGTG	G(C)CTGTA
CSSR-7	CACAGCGAACAGGCATTG	AGCAACCAAGACGGGAAC	CTGTGC
CSSR-10	GCAACCACAAAGCCGCAG	AGCACCTCTTAGCATCACTGG	CAATGA
CSSR-12	TAAGTCCATCACCGAGAAG	AAACGGATTTAGGAACACTC	GAAGGCGTA
CSSR-13	CAATGTCACTCAGGTCAG	TTCTGGAATACATCAAATGC	TGTTGGGG
CSSR-16	CGATCAACCCATTCACTG	GCT(C)CCTATTTGCATGATATTG	GTGGTGGCA
CSSR-17	AGAAGTATTCGCTACGCTACG	GGTGATGATTCAGTTGGTGTTG	CTGATGTG
CSSR-18	GTGCTTCCAGAAGTTGTG	GACTGTTCTCTTCGTTCAG	GCCAA
CSSR-19	TGCTGTGATTGGAGTTTTGC	TCAAACGAATCTGTCCATCAAG	TGGTGAG
CSSR-20	GGTATCGCCTTTGGTTCTGG	GACAACCGACATCCTCATGG	GTAGCA
ASSR-9	GGTTGTCGGGCTCATTCC	TTGTCACAGCATCACTATTCTC	CAAGTAC
ASSR-11	AGAGGCAACGCAGGAACAG	GTGAGTTATATCGGTGCAGCAG	ACGCATC
ASSR-12	TGCTCATTGTGGCGAAGG	CGCAACGTGCATTCATCG	GATTCAG
ASSR-14	TTGACTCAAGGAATAAAAC	GAAAAGAGTGTCAATACG	CTGCGTGC
ASSR-16	TTAATCAACAACGCTTATCC	TCGCAGTAGCCAGTATA(G)C	GCTCCGGTTCTA (GCTCCA)
ASSR-19	CGCCGACTGTCTATG(A)TGAC	TTCC(G)TAGCAATGGCAATGTTG	ACAACG
ASSR-20	TTACTATCGGCAGCAGACG	TGAAGCAATGGTGGATTTAGG	ACAGAAA
GSSR_4	GCGTTACTGGCGACAAA(G)C	GCTCGT(C)TCCTGACCTGTG	ATCC
GSSR_6	TGTTCTCTTCGTTCAGCCAAGC	CGCAGCAGAGCAGCAGTG	CTTGT(G)
GSSR_7	ATCATGTCGTGTCGTTTC	CAATAAAGCACCGAATTAGC	GGCAAC
GSSR_12	TTACGCTGATTGGCTGCATTG	GTCAAACACTGCCTATAGAGCG	TATCTGT
GSSR_14	TTGATGTGCTTTTGCGGTAAG	GACAGG(C)TCCTCTCATTGCG	TCCC(T)GTA
GSSR_15	CCGCAGAGTCCGTTGTA(C)AC	AGCC(T)GACGCACGGTATATC	AGCCTGC
GSSR_19	GCCGATGCAGAACAAGAAC	TCAACTTCGCCACACCTG	GAAAACAAG(C)
GSSR_20	TGGATGGATAGATGATTCAGCC	CGATCAGTGGAGGATGTCTTG	GAACCACT(C)A
**Francisco *et al.*, 2017**			
COSS1	GAAACAAGATGGCGGTTGC	CATTTAAACGGGCGGCATA	ATTGCTG
COSSR6	TGCTGCGCGATAACCAAGT	CATCCAATCAGCCCTAACCT	GTGATGCG
CSSR45	ACAGACATCACCGGCATTG	AATGTCGCTGCCAATCCAT	CACACCGAGATGGAC
COSSR4	CAAGGTGACCGCTAGCCTAT	GCTGTCATTGGGTGATGC	CAATACAC
COSSR5	ACACTGACACAACAGCCACCA	AATGGTGGGTGTGATGGTTTC	CATACAGA
COSSR3	AAGTATTCGCTACGCTACGC	GTGTGTTATGTGTGCCATTCGT	CTGATGTG
CSSR42	ATTACGCTGATTGGCTGCAT	GTTTCATTACGCGGAACAC	TGTTATC

Table S2. List of 50 VNTR loci, respective primer pairs, and TR sequence from literature. In red are reported the nucleotides that didn't correspond to the matching sequence on the DeDonno genome sequence, in green the substitutions according to the same genome. The loci selected for this study are highlighted in yellow.
