# Supplementary material for: A new inclusive MLVA assay to investigate genetic variability of Xylella fastidiosa with a specific focus on the Apulian outbreak in Italy
Source: Sci Rep. 2020 Jul 2;10:10856. doi: 10.1038/s41598-020-68072-5 (PMC7331650; doi:10.1038/s41598-020-68072-5)
Supplement: Supplementary file 4 — Supplementary information 4 [file 41598_2020_68072_MOESM4_ESM.pdf]

Sample	TR4	TR5	TR6	TR7	TR8	TR9	TR10	TR12	TR15	TR18	TR19	TR21	TR23	TR24	SSR20	SSR21	SSR28	OSSR-9	OSSR-16	OSSR-19	OSSR-20	CSSR-7	CSSR-10	CSSR-45	CSSR-18	CSSR-19	ASSR-9	ASSR-11	ASSR-12	ASSR-16	ASSR-19	GSSR-4	GSSR-7	CSSR-42	COSSR-6	COSSR-4	COSSR-5
CFBP8073	13	9	0	3	13	7	11	10	5	6	0	9	2	2	2	2	0	16	11	0	20	7	10	13	8	3	2	9	12	72	2	49	30	1	1	2	1
CFBP7970	4	3	1	3	8	10	10	4	5	3	0	12	5	2	2	0	4	11	9	5	16	12	4	8	10	3	2	8	3	61	2	24	10	7	1	2	1
CFBP8351	4	3	1	3	9	10	11	4	5	6	0	9	7	2	2	0	0	14	14	6	8	11	6	10	10	3	2	9	6	53	2	15	23	11	1	2	1
CFBP8077	13	7	4	3	4	10	4	5	5	3	0	2	1	2	7	2	0	21	13	10	5	12	3	5	7	3	2	7	7	66	2	24	17	19	1	2	1
CFBP8356	13	5	6	3	15	10	4	7	5	3	0	3	1	2	6	2	0	24	15	5	12	15	6	5	5	2	2	4	10	65	3	11	21	7	1	2	1
CFBP8419	13	4	7	3	4	10	4	12	5	3	2	3	1	2	6	2	0	13	5	3	3	15	15	5	4	2	2	4	12	66	2	11	17	21	1	2	1
CFBP8478	14	4	1	3	4	10	4	12	5	3	2	3	1	2	6	2	0	11	5	3	3	15	11	5	4	2	2	4	10	66	3	11	13	21	2	2	1
CFBP8173	13	2	1	3	9	10	7	4	5	6	6	7	1	5	12	2	0	7	8	5	1	16	2	5	22	3	3	15	7	68	12	6	23	3	1	2	1
CFBP8416	17	2	1	3	11	10	4	5	5	3	6	7	1	5	8	2	0	5	22	7	1	9	9	5	7	3	15	7	9	55	12	8	12	1	1	2	1
CFBP8417	16	2	3	3	9	10	5	6	5	3	6	3	1	3	10	2	0	4	8	10	1	9	6	5	7	4	11	12	13	65	9	17	19	1	1	2	1
CFBP8429	4	8	2	9	12	6	7	13	4	4	5	3	8	4	5	2	10	3	7	7	24	11	4	9	2	0	2	2	4	70	3	6	2	1	7	12	5
CFBP8072	4	13	2	12	14	7	4	9	6	3	6	4	1	4	7	2	5	3	12	7	1	6	5	5	11	0	2	4	14	55	2	55	2	13	9	9	1
CFBP8074	4	13	2	14	14	10	4	9	6	3	6	4	1	4	7	2	5	3	12	7	1	6	5	5	13	0	2	4	14	55	2	54	2	13	9	9	1
CFBP8402	4	7	3	27	12	9	9	16	5	3	3	3	8	4	11	2	7	3	27	12	1	9	7	5	2	1	2	2	4	47	3	6	2	7	7	10	6
CFBP8477	4	7	1	23	12	9	9	16	5	3	3	3	8	4	11	2	7	3	27	9	1	9	7	5	2	0	2	2	4	47	3	6	2	7	7	10	6
Oe_Le1	4	7	3	23	12	9	9	16	5	3	3	3	8	4	11	2	7	3	27	12	1	9	7	8	3	1	2	2	4	47	3	6	2	2	7	10	6
No_Le1	4	7	3	23	13	9	9	17	5	3	3	3	8	4	12	2	7	3	27	10	1	9	7	8	2	1	2	2	4	47	3	6	2	2	7	10	6
Oe_Le2	4	7	3	23	12	9	9	16	5	3	3	3	8	4	12	2	7	3	27	11	1	9	7	8	2	1	2	2	4	47	3	6	2	2	7	10	6
No_Le2	4	7	3	21	12	9	9	16	5	3	3	3	8	4	12	2	7	3	24	11	1	9	7	8	3	2	2	2	4	46	3	6	2	2	7	10	6
Oe_Le3	4	7	3	26	12	9	9	16	5	3	3	3	8	4	11	2	8	3	27	10	1	9	7	8	2	1	2	2	4	47	3	6	2	2	7	11	6
Pm_Le1	4	7	3	19	12	9	9	16	5	3	3	3	8	4	12	2	7	3	27	11	1	9	7	8	2	1	2	2	4	47	3	6	2	2	7	11	6
Pm_Le2	4	7	3	19	12	9	9	16	5	3	3	3	8	4	12	2	7	3	27	11	1	9	7	8	3	1	2	2	4	47	3	6	2	2	7	10	6
Pm_Le3	4	7	3	19	14	9	9	16	5	3	3	3	8	4	12	2	7	3	27	11	1	9	7	8	2	1	2	2	4	47	3	6	2	2	7	10	6
Pm_Le4	4	7	3	24	12	9	9	16	5	3	3	3	8	4	12	2	7	3	27	11	1	9	7	8	3	1	2	2	4	47	3	6	2	2	7	10	6
Oe_Le4	4	7	3	25	12	9	9	16	5	3	3	3	8	4	12	2	7	3	26	11	1	9	7	8	3	1	2	2	4	46	3	6	2	2	7	10	6
Pm_Le5	4	7	3	21	13	10	9	16	5	3	3	3	8	4	12	2	7	3	27	11	1	9	7	8	2	1	2	2	4	46	3	6	2	2	7	11	6
Pd_Le1	4	7	3	14	12	9	9	16	5	3	3	3	8	4	12	2	7	3	23	11	1	9	7	8	3	1	2	2	4	46	3	6	2	3	7	10	6
Ra_Le3	4	7	3	22	12	9	9	16	5	3	3	3	8	4	12	2	7	3	26	11	1	9	7	8	3	1	2	2	4	46	3	6	2	2	7	10	6
Ra_Le1	4	7	3	22	12	9	9	16	5	3	3	3	8	4	12	2	7	3	26	11	1	9	7	8	3	1	2	2	4	46	3	6	2	2	7	10	6
Pm_Le6	4	7	3	23	13	10	9	16	5	3	3	3	8	4	12	2	7	3	27	11	1	9	7	8	3	1	2	2	4	46	3	6	2	2	7	10	6
No_Le6	4	7	3	23	12	9	8	16	5	3	3	3	8	4	12	2	7	3	27	11	1	9	7	8	3	1	2	2	4	47	3	6	2	2	7	10	6
Oe_Le5	4	7	3	23	14	9	9	16	5	3	3	3	8	4	12	2	7	3	26	11	1	9	7	8	3	1	2	2	4	47	3	6	2	2	7	9	6
Oe_Br5	4	7	3	23	12	9	9	16	5	3	3	3	8	4	12	2	7	3	26	11	1	9	7	8	3	1	2	2	4	46	3	6	2	2	7	10	6
Oe_Br1	4	7	3	22	12	9	9	16	5	3	3	3	8	4	12	2	7	3	27	11	1	9	7	8	3	1	2	2	4	46	3	6	2	2	7	10	6
Oe_Br2	4	7	3	22	12	9	9	16	5	3	3	3	8	4	12	2	7	3	27	11	1	9	7	8	3	1	2	2	4	46	3	6	2	2	7	11	6
Oe_Br6	4	7	3	23	13	9	9	16	5	3	3	3	8	4	12	2	7	3	26	11	1	9	7	8	3	1	2	2	4	46	3	6	2	2	7	11	6
Oe_Br3	4	7	3	24	13	9	9	16	5	3	3	3	8	4	12	2	7	3	27	11	1	9	7	8	3	1	2	2	4	47	3	6	2	2	7	11	6
Oe_Ta1	4	7	3	24	12	9	9	16	5	3	3	3	8	4	12	2	7	3	24	11	1	9	7	8	3	1	2	2	4	47	3	6	2	2	7	11	6
Oe_Ta2	4	7	3	20	12	8	9	16	5	3	3	3	8	4	12	2	7	3	25	10	1	9	8	8	3	1	2	2	4	47	3	6	2	2	7	12	6
Oe_Le6	4	7	3	20	12	9	9	16	5	3	3	3	8	4	12	2	7	3	24	11	1	9	7	8	3	1	2	2	4	46	3	6	2	2	7	9	6
As_Le1	4	7	3	24	12	9	9	16	5	3	3	3	8	4	13	2	7	3	25	11	1	9	7	8	3	1	2	2	4	11	3	6	2	2	7	10	6
Ra_Le2	4	7	3	24	12	9	9	16	5	3	3	3	8	4	12	2	7	3	25	11	1	9	7	8	3	1	2	2	4	46	3	6	2	2	7	10	6
As_Le2	4	7	3	23	12	9	9	16	5	3	3	3	8	4	12	2	7	3	26	11	1	9	7	8	3	1	2	2	4	48	3	6	2	2	7	10	6
Pm_Le7	4	7	3	23	12	9	9	16	5	3	3	3	8	4	12	2	7	3	26	11	1	9	7	8	3	1	2	2	4	46	3	6	2	2	7	9	6
No_Le4	4	7	3	24	12	9	9	17	5	3	3	3	8	4	12	2	7	3	28	11	1	9	7	8	3	1	2	2	4	46	3	6	2	2	7	10	6
No_Le5	4	7	3	21	12	9	9	16	5	3	3	3	8	4	11	2	8	3	26	11	1	9	7	8	3	1	2	2	4	46	3	6	2	2	7	10	6
Ln_Le1	4	7	3	24	12	9	9	14	5	3	3	3	8	4	11	2	8	3	26	12	1	9	7	8	3	1	2	2	4	46	3	6	2	2	7	9	6
Pm_Le8	4	6	3	20	13	9	10	15	5	3	3	3	8	4	12	2	7	3	23	11	1	9	7	8	2	1	2	2	4	47	3	6	2	2	7	9	6
Pm_Le9	4	7	3	23	12	8	9	16	5	3	3	3	8	4	12	2	7	3	17	7	1	9	7	8	2	1	2	2	4	46	3	6	2	2	7	9	6
Pd_Le2	4	7	3	23	8/9	9	9	16/17	5	3	3	3	8	4	11/12	2	7	3	23	11/12	1	9	7	8	3	1	2	2	4	46	3	6	2	2	6	10	6
Pm_Le10	4	7	3	23/24	12	9	9	16	5	3	3	3	8	4	11	2	7	3	25	11	1	9	7	8	2	1	2	2	4	46	3	6	2	2	7	9	6

Table S3. Haplotypes of the 51 samples analyzed in this study. Each haplotype is constituted by an ordered series of 37 numbers indicating to the number of repetitions for the respective TR sequence. The last 2 samples, evidenced in light yellow, presented in few loci multiple amplicons corresponding to different repeat numbers, which could suggest that those plant tissues was infected by more than one haplotype contemporaneously; however, these samples were not considered in data elaboration.
