# Supplementary material for: A new inclusive MLVA assay to investigate genetic variability of Xylella fastidiosa with a specific focus on the Apulian outbreak in Italy
Source: Sci Rep. 2020 Jul 2;10:10856. doi: 10.1038/s41598-020-68072-5 (PMC7331650; doi:10.1038/s41598-020-68072-5)
Supplement: Supplementary file 5 — Supplementary information 5 [file 41598_2020_68072_MOESM5_ESM.pdf]

Locus	Repeat	Correspondence on De Donno genome			
		Position	Amplicon	Interval	Coding sequence
TR7	GCTGT	399360-399628	269	1-44	intergenic
				45-185	methyltransferase
				186-269	intergenic
TR8	GACAGG	434251-434468	218	1-153	hypothetical protein (partial)
				154-218	intergenic
TR12	GGTGTGA	557590-557835	246	1-246	intergenic
OSSR-16	CTGCTA	1779830-1780212	383	1-27	intergenic
				28-246	hypothetical protein
				247-383	intergenic
OSSR19	CAGGATCA	1417983-1418272	290	1-267	intergenic
				268-290	adenylosuccinate lyase (partial)
ASSR-16	GCTCCA	1705861-1706237	377	1-377	1,4-beta-cellobiosidase (partial)
COSSR-4	CAATACAC	824598..824910	313	1-302	hypothetical protein (partial)
				303-313	intergenic

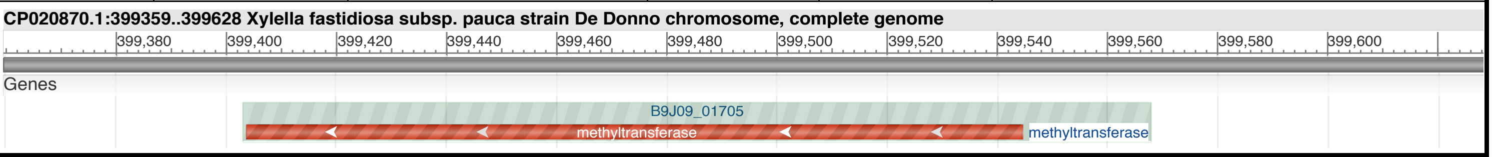

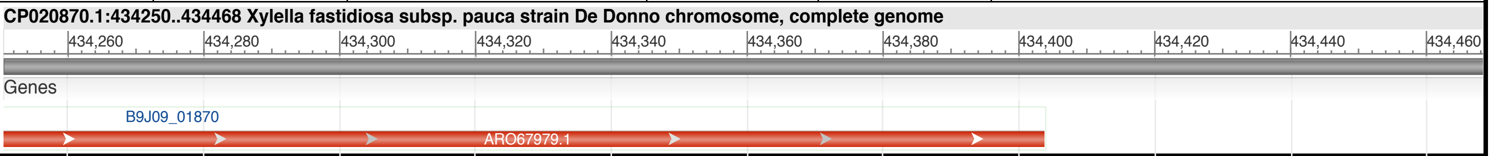

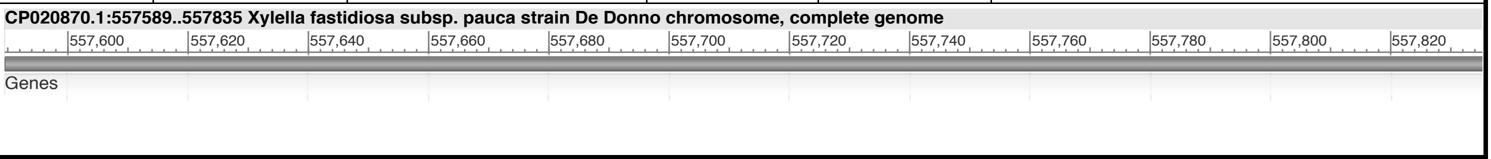

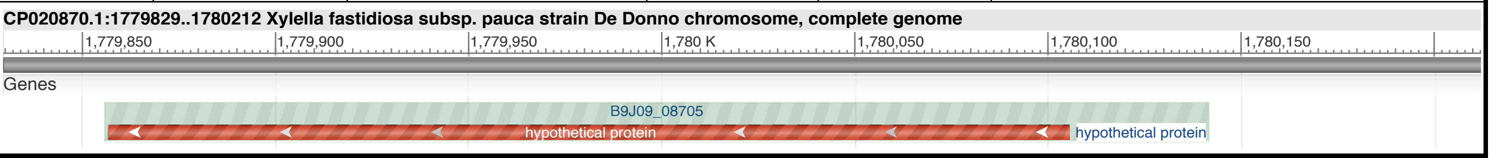

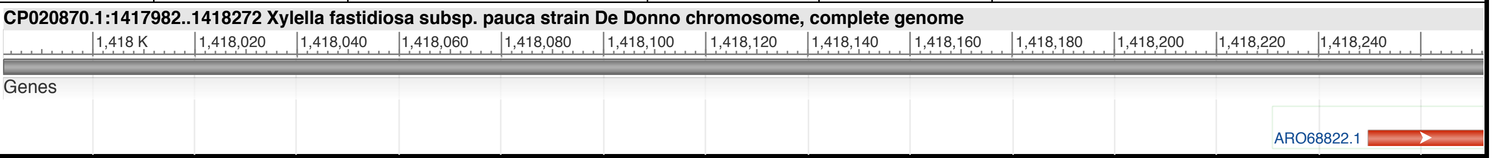

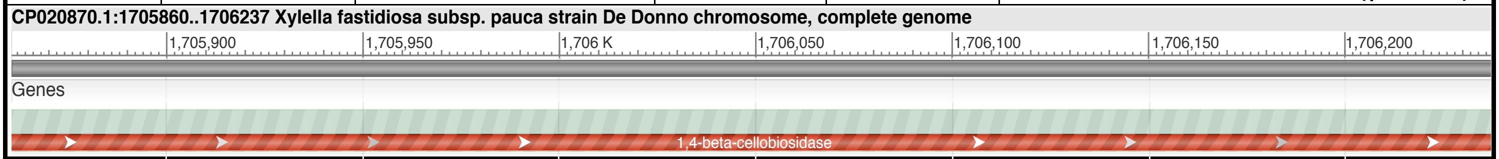

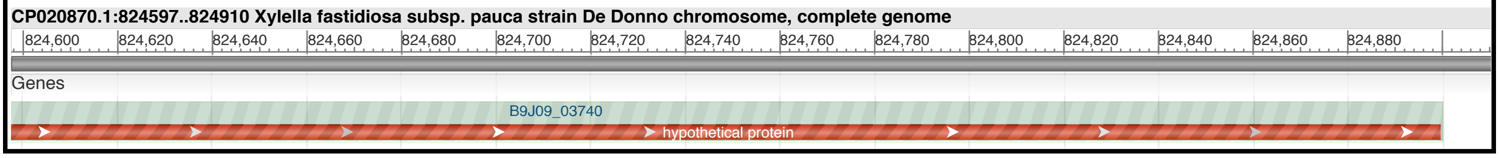

Table S4. Correspondence of the seven most variable VNTR loci, as they are amplified by the primers used here, in the genome sequence of the *Xylella fastidiosa* subspecies *pauca* strain De Donno (accession n° CP020870). The CDS are reported as deposited in NCBI database and derived by automated computational analysis using gene prediction method: Protein Homology.
